# Polymethoxyflavones and Bone Metabolism

**DOI:** 10.3390/nu17050822

**Published:** 2025-02-27

**Authors:** Michiko Hirata, Tsukasa Tominari, Chiho Matsumoto, Urara Kasuga, Keisuke Ikeda, Chisato Miyaura, Florian M. W. Grundler, Masaki Inada

**Affiliations:** 1 Department of Biotechnology and Life Science, Tokyo University of Agriculture and Technology, 2-24-16 Nakacho, Koganei-shi 184-8588, Tokyo, Japan; hirata@cc.tuat.ac.jp (M.H.); tominari@ncnp.go.jp (T.T.); c-matsu@cc.tuat.ac.jp (C.M.); miyaura@isc.chubu.ac.jp (C.M.); 2 Inada Research Unit, Institute of Global Innovation Research, Tokyo University of Agriculture and Technology, 2-24-16 Nakacho, Koganei-shi 184-8588, Tokyo, Japan; grundler@uni-bonn.de; 3 Institute of Crop Science and Resource Conservation, University of Bonn, Karlrobert-Kreiten-Strasse 13, 53115 Bonn, Germany

**Keywords:** polymethoxyflavone, osteoblast, osteoclast, bone resorption, bone metabolism, prostaglandin E2

## Abstract

Phytochemicals, such as flavonoids, are bioactive compounds produced by plants, including citrus fruits, that exhibit antioxidant effects on mammalian cells and tissues. Polymethoxyflavones (PMFs) are a family of flavonoids found in the pulp and peel of citrus fruits, and have been reported to have potent antioxidant activity implicated in the prevention of human diseases. Several studies have shown that PMFs have a protective effect on bone resorption in mouse models of diseases, including osteoporosis, rheumatoid arthritis, and periodontal disease. PMFs significantly suppressed the differentiation of osteoclasts (bone resorptive cells) through indirect and direct mechanisms. The indirect effect of PMFs is the suppression of inflammatory mediator production, such as prostaglandin E2 (PGE2), and the reduction of osteoclastic inducers, such as the receptor activator of NF-κB ligand (RANKL), in osteoblasts (bone-forming cells). The direct effect of PMF suppresses osteoclast differentiation and function by inhibiting the NF-κB signaling pathway. In silico molecular docking studies indicated that PMFs target the ATP-binding pocket of IKKβ and inhibit the NF-κB signaling pathway. These findings suggest that PMFs protect against bone destruction by interfering with the NF-κB pathway in osteoblasts and osteoclasts. In this review, we summarize the latest findings regarding the effects of PMFs on various bone resorption-related diseases in mouse models.

## 1. Introduction

Bone remodeling is a dynamic process precisely regulated by the balance between osteoclastic bone resorption and osteoblastic bone formation [1]. Several factors, such as inflammatory responses, hormone imbalance, mechanical stress, and aging, negatively regulate bone remodeling. Activation of osteoclastic bone resorption is a significant cause of bone-related diseases characterized by decreased bone mineral density (BMD), such as rheumatoid arthritis, osteoporosis, and periodontal disease [2]. Osteoclasts are multinucleated cells that are formed by differentiation and fusion from the hematopoietic cell lineage and have the unique ability to resorb bone. Osteoblasts are bone-forming cells responsible for the secretion of the bone matrix and subsequent bone mineralization, while also supporting osteoclast differentiation and function through the expression of receptor activator of NF-κB ligand (RANKL), making it a unique system in bone remodeling. RANKL, a member of the tumor necrosis factor (TNF) family, is recognized by RANK, a member of the TNF-receptor family, expressed in osteoclast precursor cells, and activates key transcription factors downstream of RANK signaling, such as nuclear factor of activated T cells 1 (NFATc1), NF-κB, and activator protein (AP)-1, leading to the differentiation of osteoclasts from osteoclast precursor cells [3]. Inflammatory molecules such as interleukins (ILs), TNF, and lipopolysaccharide (LPS) activate the NF-κB and AP-1 pathways and upregulate the expression of RANKL in osteoblasts. Therefore, blocking these transcription factors is a potential therapeutic target for bone-related diseases because of the increased differentiation and function of osteoclasts. The anti-RANKL antibody is clinically used in the treatment of osteoporosis, and several drugs targeting TNF are also used for the treatment of rheumatoid arthritis.

Phytochemicals are biologically active compounds, including carotenoids and flavonoids, derived from plants, which have various beneficial effects on human health. Incorporating phytochemicals into a nutritious diet of foods and related supplements has become a current trend in the prevention of lifestyle-related diseases in a super-aged society. In this review, we summarize the latest findings regarding the effects of PMFs on bone metabolism.

## 2. Strategy of Reviewing Criteria for Biological Activities for PMFs

We systematically searched for research articles published up to February 2025 using the PubMed, Web of Science, and Google Scholar databases. The ClinicalTrials.gov registry was searched for human studies. The search terms were as follows: polymethoxyflavone, PMFs, nobiletin, tangeretin, sinensetin, sudachitin, osteoporosis, rheumatoid arthritis, osteoarthritis, periodontal disease, and bone diseases. The inclusion criteria were as follows: research articles published in any language, publication year, animal models or humans, and administration route of PMFs. The exclusion criteria were as follows: (1) inappropriate results; (2) specific article types, including systematic reviews, notes, and commentary; and (3) written in a language other than English.

## 3. Polymethoxyflavone

**Figure 1:**
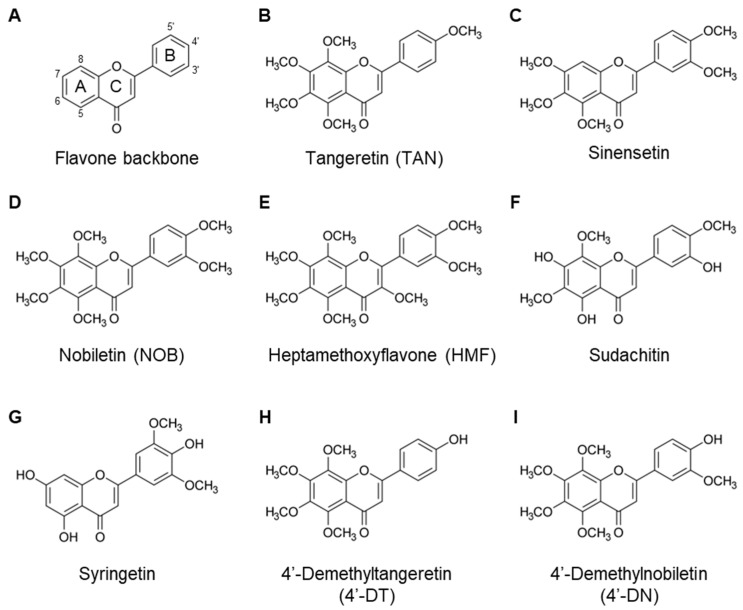
Chemical structures of PMFs. The flavone backbone (**A**). Several PMFs were identified, as follows: (**B**) Tangeretin. (**C**) Sinensetin. (**D**) Nobiletin. (**E**) Heptamethoxyflavone. (**F**) Sudachitin. (**G**) Syringetin. (**H**) 4′-Demethyltangeretin. (**I**) 4′-Demethylnobiletin.

There are three major groups of citrus flavonoids: flavanones, flavone glycosides, and PMFs. PMFs are a unique family of flavonoids found in citrus fruit peels, with multiple methoxy groups on their chemical flavone backbone (Figure 1A), such as tangeretin (4′,5,6,7,8-pentamethoxyflavone), sinensetin (3′,4′,5,6,7-pentamethoxyflavone), nobiletin (3′,4′,5,6,7,8-hexamethoxyflavone), and Sudachitin (4′,5,7-trihydroxy-3′,6,8-trimethoxyflavone) (Table 1 and Figure 1). They have been extensively studied for their beneficial effects on human health, including anti-oxidative, anti-inflammatory, and anti-obesity effects [4,5,6]. Differences in the number and position of the methoxy groups influence their biological activity. We previously reported that several flavonoids, including nobiletin [7,8], tangeretin [8], 3,5,6,7,8,3′,4′-heptamethoxyflavone (HMF) [9], and demethylated metabolites [10], exhibit anti-bone resorptive effects in mice. 

## 4. Prostaglandin E_2_ as a Key Mediator of Inflammatory Bone Resorption

**Figure 2:**
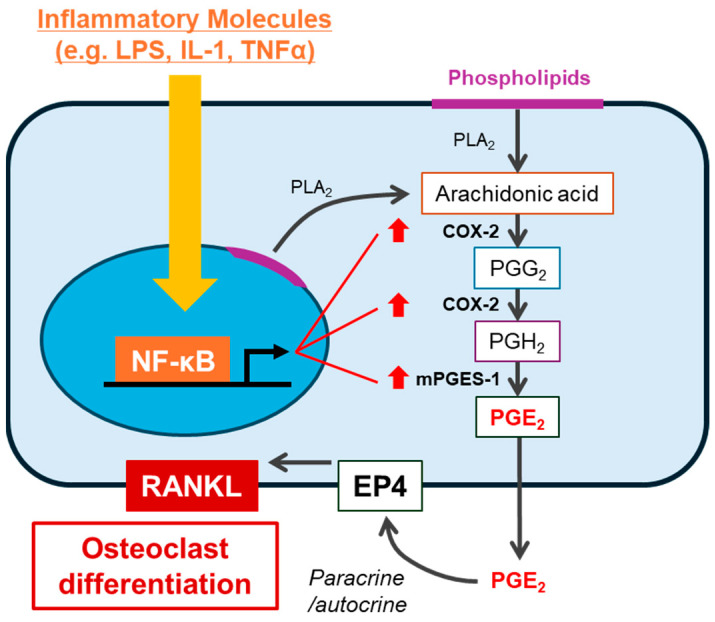
Bone resorptive molecules stimulate PGE_2_-mediated bone resorption. Several molecules (e.g., LPS, IL-1, and TNFα) activate the NF-κB pathway in osteoblasts. NF-κB transcriptionally upregulates PGE synthases, including COX-2 and mPGES-1, leading to the production of PGE_2_. Subsequently, the autocrine/paracrine effect of PGE_2_ via EP4 enhances the expression of RANKL, leading to osteoclast differentiation.

Prostaglandins (PG) are lipid mediators that act in an autocrine and/or paracrine manner via their own G-protein coupled receptors (GPCRs), respectively. PGs mainly consist of PGE_2_, PGD_2_, PGF_2α_, and PGI_2_. PGE_2_ is the best studied osteoclastogenic mediator in bone-related diseases. PGE_2_ is synthesized via an arachidonic acid (AA) cascade [11], which is released from membrane phospholipids by phospholipase A_2_ and converted into PGH_2_ by cyclooxygenases (COXs). PGE_2_ is synthesized from PGH_2_ by PGE synthase (PGESs). In bone tissues, inflammatory responses by cytokines, including IL-1 and tumor necrosis factor-α (TNF-α), stimulate the expression of COX-2 and membrane-type PGE synthase-1 (mPGES-1), which are inducible enzymes, resulting in the overproduction of PGE_2_ and subsequent induction of RANKL expression on the surface of osteoblasts (Figure 2) [12,13]. In periodontal disease, bacterial components, such as lipopolysaccharide (LPS), induce alveolar bone resorption by stimulating PGE_2_ production [14,15]. Inhibition of PGE_2_ synthesis by treatment with indomethacin (a non-selective COX inhibitor) or blocking PGE_2_ receptor (EP) signaling by treatment with an EP antagonist or knocking out membrane-bound PGE synthase inhibits LPS-induced alveolar bone resorption in mice [16,17]. PGE_2_ has also been shown to have high concentrations in the synovial fluid of patients with RA and osteoarthritis (OA), and participates in joint destruction and pain [18,19].

PGE receptors EP1–4 are GPCRs [20]. Osteoblasts express these four receptors; however, EP2 agonists and EP4 agonists, but not agonists of EP1 and EP3, stimulate osteoclast differentiation associated with the increased expression of RANKL [16]. On the other hand, in bone cultures, PGE_2_-induced bone resorption was abolished in EP4^−/−^ mice, but not in EP1^−/−^, EP2^−/−^, and EP3^−/−^ mice [21]. Thus, prostaglandin E_2_ (PGE_2_) is a critical bone resorptive molecule involved in bone resorption induced by LPS or other cytokines via EP4 (possibly partially involving EP2) (Figure 2).

## 5. Effects of PMFs on Bone Metabolism

### 5.1. Nobiletin

Nobiletin, a PMF with six methoxy groups, is abundant in citrus peels, especially those of *Citrus depressa*, and its role in bone metabolism has been well studied. Our previous studies have shown that nobiletin significantly suppresses osteoclast differentiation and bone-resorbing activity [7,8]. Treatment with 10, 30, and 60 μM nobiletin inhibited IL-1-induced osteoclast differentiation by 20, 85, and 100%, respectively, in co-cultures of osteoblasts and bone marrow cells. Treatment with 60 μM nobiletin completely suppressed IL-1-induced bone resorption in mouse bone organ cultures. In addition, LPS-induced osteoclast differentiation and bone-resorbing activity were significantly suppressed by 30 μM nobiletin in the co-cultures and bone organ cultures. Mechanistically, nobiletin suppressed PGE_2_ production associated with the downregulation of COX-2, a target gene of NF-κB, in osteoblasts. An in vitro kinase assay revealed that nobiletin directly inhibited IKKβ kinase activity. In addition, the nuclear translocation and transcriptional activity of NF-κB were suppressed by nobiletin. These data indicate that nobiletin inhibits IKK-dependent NF-κB activation and suppresses COX-2-mediated PGE_2_ production in osteoblasts, resulting in downregulation of RANKL expression and attenuation of RANKL/RANK signaling in osteoclast precursor cells [7,8]. As RANKL-RANK signaling activates the IKK-dependent NF-κB pathway, NF-κB blockage by nobiletin contributes to the direct inhibition of RANKL-induced osteoclast differentiation in the murine osteoclast precursor cell line Raw264.7 cultures [8]. Murakami et al. showed that nobiletin (4, 20, and 50 μM) suppressed RANKL-induced osteoclast differentiation in a dose-dependent manner by inhibiting the phosphorylation of MAPKs, including ERK1/2, JNK1/2, and p38, and the degradation of IκBα protein, which in turn blocked the activation of AP-1 and NF-κB in Raw264.7 [22]. On the other hand, several studies have reported that nobiletin stimulates bone formation. Pang et al. reported that 10 and 20 μg/mL (approximately 25 and 50 μM, respectively) of nobiletin promoted osteoblast differentiation and mineralization in a dose-dependent manner by activating the bone morphogenetic protein-2 (BMP-2)/runt-related transcription factor-2 (RUNX-2) pathway in the human osteoblastic cell line MG-63 [23]. Rojasawasthien et al. reported that implantation of a collagen sponge disk containing BMP-2 (1 μg) and nobiletin (2.5 or 5 μg) into the fascia of the back muscle in mice induces BMP-22-induced ectopic bone formation by inhibiting NF-κB activity and subsequently enhancing BMP signaling [24]. Nobiletin increased bone formation (BV/TV) by 33% relative to BMP-2 alone. These data indicate that nobiletin has a dual effect on the negative regulation of bone resorption and positive regulation of bone formation.

As an effect of nobiletin on postmenopausal osteoporosis, we and other groups demonstrated that nobiletin prevents estrogen deficiency-induced bone loss without affecting body weight and the uterus in a postmenopausal osteoporosis model, ovariectomized (OVX) mice [7,22,25]. In our previous study, ovariectomized (OVX) mice exhibited a 67% decrease in bone volume/tissue volume (BV/TV) and a 13% reduction in bone mineral density (BMD) of the femur compared to Sham mice, while intraperitoneal administration of nobiletin at 2 mg/mouse/day (approximately 67 mg/kg) for 4 weeks restored these bone parameters by 34% and 6%, respectively (using BMD in Sham mice as 100%) [7]. Murakami et al. showed that the subcutaneous administration of nobiletin (0.25 mg/day delivered by a mini-osmotic pump installed subcutaneously for 4 weeks in OVX mice had 15% less distal BMD and 9% less whole BMD in the femur relative to sham mice, while these BMD values were restored by 5% and 4%, respectively, by nobiletin treatment [22]. Lee et al. tested the effects of the oral administration of 50 and 100 mg/kg nobiletin for 12 weeks in OVX mice. OVX mice showed 17% less trabecular BMD in the distal femur, whereas treatment with nobiletin restored BMD by 8%, with similar effects at both dosages [25]. Notably, Wang et al. developed nobiletin-loaded poly(ethylene glycol)-block-poly(e-caprolactone) micelles (particle size: 124 nm) as a drug delivery system (DDS) to improve the solubility and stability of nobiletin and enhance its bone-targeted delivery [26]. They demonstrated that nobiletin without micelles, but not nobiletin-loaded micelles, was cytotoxic to bone marrow macrophages (BMMs) at a concentration of 50 μM. In addition, nobiletin-loaded micelles, at a concentration 50 μM, effectively suppressed RANKL-induced osteoclast differentiation by 83%. This was associated with downregulation of the expression of osteoclast marker genes, including tartrate-resistant acid phosphatase and cathepsin K, and a decrease in phosphorylation of MAPKs in BMM cultures. Their study showed that OVX mice exhibited 56% reduction in BV/TV, 8% reduction in BMD, and 76% increase in trabecular separation (Tb.Sp). The intraperitoneal administration of 50 μM nobiletin without carriers or without nobiletin-loaded micelles for 9 weeks resulted in improvements in bone parameters. Specifically, BV/TV was restored by 16% without carriers and 28% without nobiletin-loaded micelles, BMD was restored by 4.5% without carriers and 5.6% without nobiletin-loaded micelles, and Tb.Sp was restored by 30% without carriers and 59% without nobiletin-loaded micelles.

Hosokawa et al. reported that nobiletin (12.5, 25, 50, and 100 μM) dose-dependently reduced the expression of inflammatory cytokines (CXCL10, CCL2, IL-8) and matrix metalloproteinases (MMPs), including MMP-1, MMP-3, and COX-2 in TNF- or IL-1-stimulated human periodontal ligament cells (HPDLCs) [27,28]. These inhibitory effects of nobiletin are mediated by suppression of the MAPK, NF-κB, and AKT pathways activated by TNF or IL-1. Our previous study showed that the local injection of LPS (25 μg/mouse/day, three times every other day) into periodontal tissue decreased alveolar bone mineral density (ABMD) by 9% in mice, while the co-injection of LPS and nobiletin (3, 100, and 300 μg/mouse/day, three times every other day) decreased ABMD by 3%, 4%, and 1%, respectively, suggesting that nobiletin inhibits LPS-induced alveolar bone resorption [8]. These reports indicate that the anti-inflammatory properties of nobiletin as well as its anti-osteoclastogenic effects contribute to its inhibitory effects on periodontal bone resorption.

Nobiletin has the potential to mitigate inflammatory bone destruction in arthritis. In collagen-induced arthritic mice, nobiletin has been reported to downregulate the gene expression and production of aggrecanase-1 and -2 [29], and the intraperitoneal administration of nobiletin (15, 30, and 60 mg/kg) decreased the severity of arthritis by 45% [22]. Liu et al. reported that 50 μM nobiletin suppressed the IL-21-mediated inflammatory response in MH7A human fibroblast-like synoviocytes (FLS) [30]. Xie et al. reported that nobiletin significantly suppressed the overproduction of inflammatory mediators, such as PGE_2_, nitric oxide (NO), COX-2, inducible NO synthase (iNOS), TNF-α, IL-6, and MMPs in IL-1-stimulated human OA chondrocytes, and prevented cartilage destruction and thickening of the subchondral bone in destabilization of the medial meniscus (DMM)-OA model mice [31]. Lin et al. reported that nobiletin (10, 20, and 40 μM) inhibited IL-1-induced NF-κB signaling in a dose-dependent manner and suppressed the production of inflammatory and catabolic mediators, including MMPs, iNOS, and COX-2, in chondrocytes [32]. In addition, the results showed that the intraperitoneal injection of nobiletin at 20 mg/kg every 2 days for 8 weeks inhibited cartilage destruction and synovitis in DMM-OA mice, suggesting that nobiletin has a positive effect on ameliorating bone destruction in arthritis.

### 5.2. Tangeretin

Tangeretin is a PMF with five methoxy groups found in citrus peels. We previously reported that 30 μM tangeretin significantly suppressed osteoclast differentiation by 70–90% induced by IL-1, LPS, or soluble RANKL (sRANKL) by inhibiting IKK-dependent NF-κB activation and subsequent PGE_2_ production [8]. Similar to nobiletin, the co-injection of LPS and nobiletin (3, 100, and 300 μg/mouse/day, three times every other day) reduced ABMD by 3%, 4%, and 1%, respectively, compared to a 9% reduction in ABMD by LPS, although the effect of tangeretin was less than that of nobiletin [8]. Tangeretin can ameliorate bone destruction in arthritis. Li et al. showed that tangeretin (50 and 100 μM) significantly inhibits the proliferation of rheumatoid synovial fibroblasts (RASFs) and downregulates the expression of MMPs and COX-2 by reducing the expression of NF-κB [33]. Li et al. reported that daily oral administration of tangeretin for 14 days reduced oxidative stress and inflammation by upregulating Nrf2 signaling in CIA rats [34]. These reports suggest that tangeretin potentially ameliorates bone destruction in arthritis by downregulating NF-κB and upregulating Nrf2 signaling.

### 5.3. Sinensetin

Sinensetin contains five methoxy groups found in *Citrus myrtifolia, Citrus leiocarpa,* and *Orthosiphon aristatus* var. Yuan et al. reported that sinensetin (2.5, 5, and 10 μM) dose-dependently reduced oxidative stress and inflammation in periodontal ligament cells and that oral administration of sinensetin (5, 10, and 20 mg/kg/day) for 3 weeks suppressed alveolar bone resorption in rats with ligature-induced periodontitis [35]. Mechanistically, they showed that Bach1 is a key target of sinensetin, which directly binds to the transcription factor BTB and CNC homology 1 (BACH1) and promotes its ubiquitination and degradation, leading to the upregulation of the antioxidative factor heme oxygenase-1 (HO-1) and its antioxidative activity.

Liu et al. indicated that sinensetin delays the progression of OA, possibly by attenuating the overproduction of inflammatory mediators, such as COX-2, iNOS, TNF-α, and IL-6, through upregulation of serpin family A member 3 (SERPINA3) expression and subsequent suppression of the NF-κB pathway [36]. Zhou et al. showed that sinensetin promotes autophagy via AMPK/mTOR signaling, inhibits apoptosis, reduces MMP13 production, and promotes aggrecan and collagen II production in chondrocytes [37]. They also showed that the oral administration of sinensetin at a dose of 50 mg/kg/day for 8 weeks protects against disease progression in DMM-OA mice.

### 5.4. Other PMFs and Demethylated PMF Metabolites

Other PMSs and their metabolites have been reported to exert direct or indirect beneficial effects on bone tissues. Manthey et al. reported that intraperitoneal administration of HMF possessing six methoxy groups (100 mg/kg), but not oral administration (100 mg/kg), reduced serum TNF-α levels in carrageenan-induced paw edema in rats [38]. Okuyama et al. reported that the subcutaneous administration of HMF (100 mg/kg) downregulates IL-1β mRNA expression in the hippocampus of mice injected intrahippocampally with LPS [39]. We previously reported that 30 μM HMF inhibited LPS-induced bone resorption by >90% in mouse calvarial or alveolar bone organ cultures [9]. PMF mixtures consisting of nobiletin, tangeretin, HMF, and 4′,5,6,7-tetramethoxyflavone (35.7%, 11.0%, 2.4%, and 38.8%, respectively) exhibited significant inhibitory effects on osteoclast differentiation and bone resorption. Oral administration of PMF mixtures at 5 mg/mouse/day (approx. 167 mg/kg) for 4 weeks prevented bone loss in OVX mice, and local co-injection of LPS and PMF mixtures suppressed alveolar bone loss in mice [40].

Ohyama et al. reported that Sudachitin (2, 10, and 50 μM), a PMF with three hydroxy and three methoxy groups found in the peel of *Citrus Sudachi*, dose-dependently suppressed RANKL-induced osteoclast differentiation by reducing reactive oxygen species (ROS) production and attenuating MAPK pathways in bone marrow macrophage (BMM) cultures [41]. Hosokawa et al. reported that sudachitin reduced the production of matrix metalloproteinase (MMP)-1 and MMP-3 by inhibiting the TNF-α/AKT pathway in human periodontal ligament cells [42].

Tsai et al. reported that 20 μM syringetin exhibited the strongest inhibitory effect on M-CSF-/RANKL-induced osteoclast differentiation compared to 20 μM laricitrin (3,3′,4′,5,7-Pentahydroxy-5′-methoxyflavone) and 20 μM myricetin (3,3′,4′,5,5′,7-Hexahydroxyflavone). In addition, syringetin inhibited the production of M-CSF and RANKL and promoted the production of osteoprotegerin (OPG), a decoy receptor for RANKL, in osteoblasts stimulated by lung cancer cells, thereby blocking the AKT-mTOR pathway and suppressing osteoclast differentiation [43].

**Figure 3:**
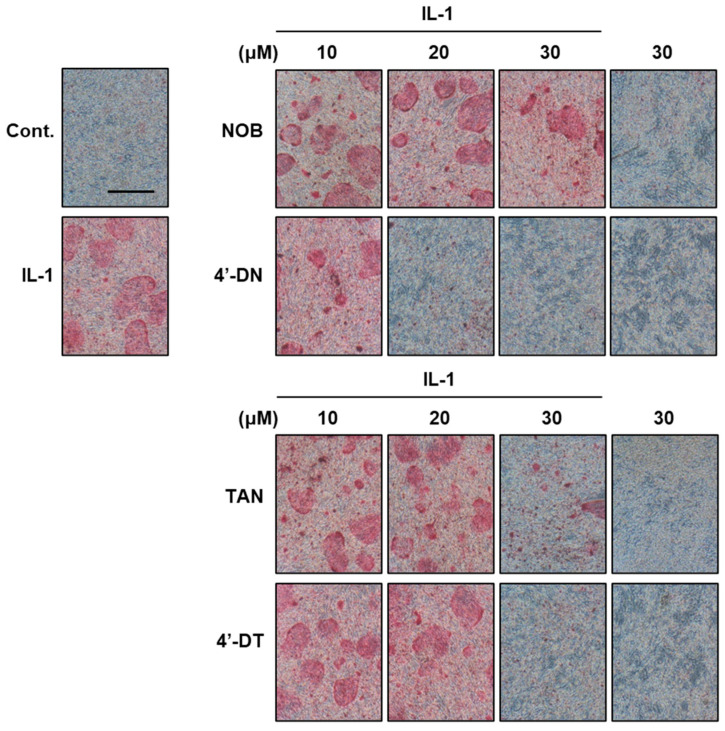
Effects of 4′-demethylnobiletin and 4′-demethyltangeretin on osteoclast differentiation. Images show TRAP-positive osteoclasts. The compounds 4′-DN and 4′-DT exhibited potent inhibitory effects on IL-1-induced osteoclast differentiation relative to nobiletin and tangeretin, respectively. These data were obtained from a previous study: Hirata M, Tominari T, and Inada M, et al. Effects of 4′-Demethylnobiletin and 4′-Demethyltangeretin on Osteoclast Differentiation In Vitro and in a Mouse Model of Estrogen-Deficient Bone Resorption [10].

**Figure 4:**
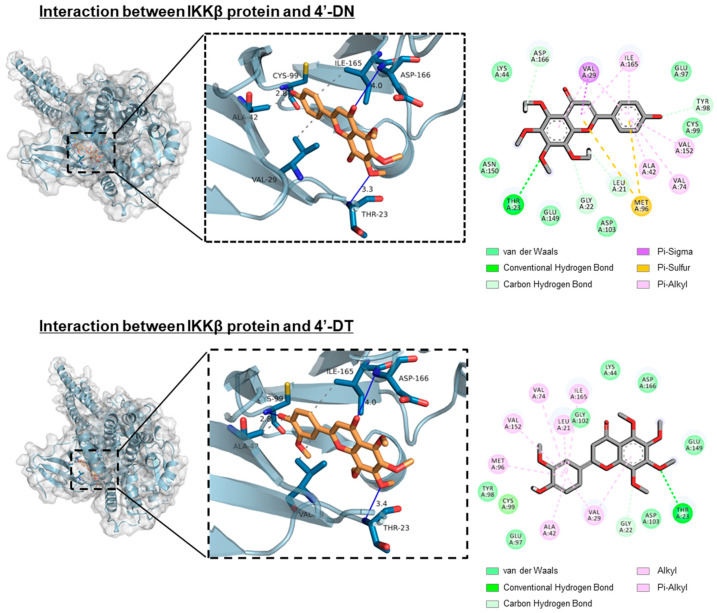
Docking simulation between IKKβ protein and 4′-DN or 4′-DT. In silico molecular docking test was performed using AutoDock Vina. Three-dimensional docking models of IKKβ protein and 4′-DN (upper) or 4′-DT (lower). These data were obtained from a previous report: Hirata M, Tominari T, and Inada M, et al. Effects of 4′-Demethylnobiletin and 4′-Demethyltangeretin on Osteoclast Differentiation In Vitro and a Mouse Model of Estrogen-Deficient Bone Resorption [10].

Recently, demethylated metabolites of PMF were reported to possess stronger bioactivity than the original compounds. Li et al. showed that 4′-demethylnobiletin (4′-DN) and 3′,4′-DN exert potent anti-inflammatory activities compared to nobiletin [44]. Wang et al. reported that 5-DN and 5-DT exerted more potent antioxidant activities than nobiletin and tangeretin [45]. The compound 4′-DN and 4′-demethyltangeretin (4′-DT) suppressed IL-1-or RANKL, showing a higher inhibitory activity than nobiletin and tangeretin (Figure 3) [10]. Similar to nobiletin, 4′-DN and 4′-DT attenuated the NF-κB pathway by binding to the ATP pocket of the IKKβ protein (Figure 4). Intraperitoneal administration of a mixture of 4′-DN and 4′-DT at 2 mg/mouse/day (approximately 67 mg/kg) for 4 weeks significantly protected OVX mice against bone loss.

## 6. Relationship Between Chemical Structure and Biological Activities of PMF

The chemical structure–biological activity relationship (SAR) is an important aspect of understanding the biological effects of phytochemicals on human health. The presence, number, and position of the methoxy groups can modulate the biological activity of PMFs. Kawaii et al. reported the anti-proliferative effects of 20 PMFs on HL-60 cells [46]. They indicated that an increase in the number of methoxy groups on the B-ring moiety of PMFs with the same A-ring methoxy group patterns tended to decrease the activity (tangeretin vs. nobiletin or heptamethoxyflavone). In contrast, an increase in the number of methoxy groups on the A-ring moiety enhanced activity. The position rather than the number of methoxy groups on the B-ring moiety affected the activity. Furthermore, the presence of a hydroxy group at the C3-position of PMFs with the same methoxy group patterns on the A- and B-ring moieties strongly enhanced the activity (natsudaidain [3′,4′,5,6,7,8-Hexamethoxy-3-hydroxyflavone] vs. nobiletin or heptamethoxyflavone).

Lam et al. reported the antiproliferative effects of flavonoids, including sinensetin (five methoxy groups), nobiletin (six methoxy groups), scutellarein tetramethylether (four methoxy groups), scutellarein (four hydroxy groups), and hesperetin (one methoxy group and three hydroxy groups) on human umbilical vein endothelial cells (HUVECs) [47]. The IC50 (50% inhibitory concentration) for the antiproliferative effect of these flavonoids on HUVECs was 24 μM for sinensetin, 60 μM for nobiletin, >100 μM for hesperetin, >100 μM for scutellarein tetramethylether, and >100 μM for scutellarein (sinensetin > nobiletin > hesperetin > scutellarein tetramethylether > scutellarein). In addition, the anti-angiogenic activities of sinensetin, nobiletin, and hesperitin at 30 μM in zebrafish were approximately 95%, 63%, and 30%, respectively. For the angiogenic aspects of PMFs, the presence of the methoxy group at the C3′-position (sinensetin and nobiletin vs. hesperetin) and the absence of the methoxy group at the C8-position (sinensetin vs. nobiletin) enhanced anti-angiogenic activity.

We previously compared the effects of nobiletin, tangeretin, and their demethylated metabolites [8,10]. The inhibitory effect of nobiletin is lower than that of tangeretin in co-cultures and bone organ cultures, but higher than that of tangeretin in Raw264.7 cultures (Table 2). The demethylated metabolites showed stronger activity than the original PMFs. In addition, demethylated nobiletin showed greater inhibitory effects than demethylated tangeretin in both co-cultures and Raw264.7 cultures. Regarding anti-osteoclastogenic effects, the absence of a methoxy group at the C3′-position (nobiletin vs. tangeretin) may slightly enhance the anti-osteoclastogenic activity, whereas nobiletin inhibits alveolar bone loss by LPS more strongly than tangeretin in vivo. The absence of a methoxy group at the C3′-position, combined with the presence of a hydroxy group at the C4′-position (4′-DN vs. 4′-DT) decreased the activity. Substitution of the methoxy group with the hydroxy group at the C4′-position (nobiletin vs. 4′-DN, tangeretin vs. 4′-DT) enhances anti-osteoclastogenic activity. Thus, several factors, including the presence or absence, number, and combination of methoxy and other functional groups, as well as target cells, influence the activity of PMFs.

## 7. PMFs for Human Clinical Studies

Some human clinical studies have been conducted on PMFs. Yoshino et al. reported the results of a randomized, double-blind, placebo-controlled, parallel group study of PMFs from *Kaempferia parvifloa* in overweight Japanese individuals (*n* = 38–39) (UMIN Clinical Trials Registry: UMIN000037453) [48]. The study showed that visceral and subcutaneous fat areas were reduced after 12 weeks in the active group supplemented with PMFs (12 mg/day) relative to the placebo group, without any adverse events. Yamada et al. reported the effects of nobiletin-rich test foods on the cognitive function of elderly Japanese individuals [49]. The intake of nobiletin-rich test food at 30 mg nobiletin and 17.4 mg tangeretin per day for 16 weeks (*n* = 54) resulted in a significant improvement in the memory function, as assessed by Wechsler Memory Scaled-Revised (WMS-R) scores, in comparison to the placebo control (*n* = 54), without adverse events.

An anticipated adverse effect of long-term flavonoid supplementation is an estrogen-like effect. Although isoflavones, such as genistein and daidzein, have estrogenic effects via binding to estrogen receptors [50], there are no reports on polymethoxyflavones. We confirmed that among PMFs, nobiletin is unable to bind to estrogen receptor-α/-β using an estrogen receptor-binding assay (unpublished data). On the other hand, excessive long-term intake of PMFs may indicate some toxicity, since a high concentration of PMFs may lead to inhibited cell growth or cell death in various cell types, including osteoblasts, endothelial cells [47], and cancer cell lines [51,52,53].

Regarding the bioavailability of PMFs, as mentioned above, the intake of PMFs at 12 mg/day reduced visceral fat in overweight Japanese individuals, indicating that dietary PMFs are absorbed in the intestines and circulate through the body in the bloodstream. However, it remains unclear how much of these compounds actually reach the bones [48]. The utilization of DDS, such as nobiletin-loaded micelles, as reported by Wang et al. [26], is a promising strategy for delivering PMF to bones. DDS has the potential to achieve effective delivery of PMFs to bones by nanocarriers conjugated with antibodies and/or enzymes targeting bone cell-specific molecules [54], calcium-based nanomaterials [55], and bisphosphonate-modified nanoparticles [56]. Further studies are needed to determine the criteria for safety, dosage, synergistic efficacy with other compounds, bioavailability, as well as the adverse effects of PMFs in animal models and human trials for preventing human bone diseases.

## 8. Conclusions and Future Perspectives

**Figure 5:**
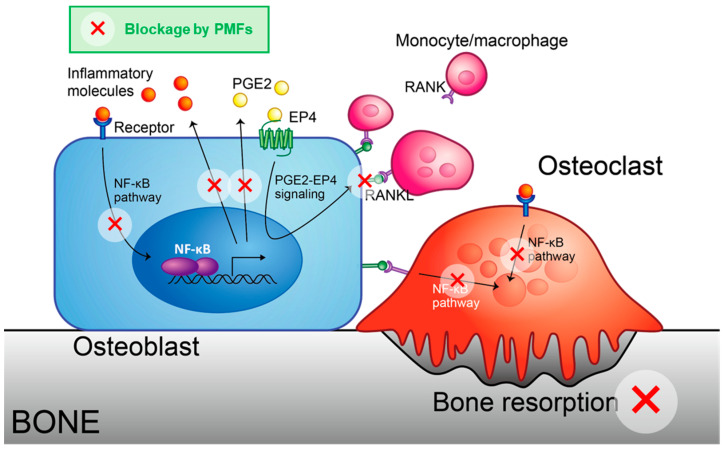
Schematic model for the inhibition of osteoclast differentiation by PMFs. Hydrophobic PMFs can cross the cell membrane and bind to IKKβ protein, blocking the NF-κB pathway. Thus, PMFs attenuated NF-κB-driven expression of inflammatory cytokines and PGE synthases, thereby decreasing the expression of RANKL.

This review summarizes the latest findings regarding the effects of PMFs on bone metabolism. PMFs act on osteoblasts, osteoclast precursor cells, and mature osteoclasts, thereby inhibiting osteoclast differentiation and function (Figure 5). Thus far, these results suggest that PMFs acts by inhibiting NF-κB on osteoblasts, which are responsible for bone formation, and on osteoclasts by producing RANKL. PMFs were shown to prevent bone loss in vivo in a mouse model of bone resorptive diseases as a preclinical proof-of-concept; however, to consider its application to humans as a PMF-containing dietary supplement, there are several issues that need to be addressed.

In conclusion, PMFs are dietary supplements that may prevent bone-related diseases. Further studies are needed to investigate the blood concentrations of PMFs and their distribution in bone. PMFs, historically consumed from citrus fruits, have the potential to prevent bone-resorptive diseases and maintain bone health.

## Figures and Tables

**Table 1:** Chemical structures of polymethoxyflavones.

Name	Synonyms	Residues
Tangeretin	4′,5,6,7,8-Pentamethoxyflavone	5 methoxy groups
Sinensetin	3′,4′,5,6,7-Pentamethoxyflavone	5 methoxy groups
Nobiletin	3′,4′,5,6,7,8-Hexamethoxyflavone	6 methoxy groups
Heptamethoxyflavone	3,3′,4′,5,6,7,8,-Heptamethoxyflavone	7 methoxy groups
Sudachitin	4′,5,7-Trihydroxy-3′,6,8-trimethoxyflavone	3 methoxy groups 3 hydroxy groups
Syringetin	3,4′,5,7-Tetrahydroxy-3′,5′-dimethoxyflavone	2 methoxy groups 4 hydroxy groups
4′-Demethyltangeretin	4′-Hydroxy-5,6,7,8-Tetramethoxyflavone	4 methoxy groups 1 hydroxy groups
4′-Demethylnobiletin	4′-Hydroxy-3′,5,6,7,8-Pentamethoxyflavone	5 methoxy groups 1 hydroxy groups

**Table 2:** Structure–activity relationships of polymethoxyflavones.

Target	PMFs		Effects	
Osteoclast differentiation in co-culture system	IL-1 +	Nobi	33 μM	50% (IC50) [10]
		DeNobi	13 μM	50% (IC50) [10]
		Tang	18 μM	50% (IC50) [10]
		DeTang	18 μM	50% (IC50) [10]
	LPS +	Nobi	30 μM	70% inhibited [8]
		Tang	30 μM	94% inhibited [8]
Osteoclast differentiation in Raw264.7 cultures	RANKL +	Nobi	8 μM	50% (IC50) [10]
		DeNobi	1 μM	50% (IC50) [10]
		Tang	16 μM	50% (IC50) [10]
		DeTang	7 μM	50% (IC50) [10]
	RANKL +	Nobi	30 μM	86% inhibited [8]
		Tang	30 μM	71% inhibited [8]
Alveolar bone loss in mice	LPS + Nobiletin		30 μg/mouse	5% restored [8]
			100 μg/mouse	6% restored [8]
			300 μg/mouse	9% restored [8]
	LPS + Tangeretin		30 μg/mouse	0% restored [8]
			100 μg/mouse	2% restored [8]
			500 μg/mouse	4% restored [8]
Anti-proliferative effect on HL-60 cells	Sinensetin		>400 μM	50% (IC50) [46]
	Nobiletin		52 μM	50% (IC50) [46]
	Tangeretin		32 μM	50% (IC50) [46]
	Heptamethoxy flavone		63 μM	50% (IC50) [46]
	Natsudaidain		5 μM	50% (IC50) [46]
Anti-proliferative effect on HUVECs	Sinensetin		24 μM	50% (IC50) [47]
	Nobiletin		62 μM	50% (IC50) [47]
	Hesperetin		>100 μM	50% (IC50) [47]
	Scutellarein		>100 μM	50% (IC50) [47]
	Scutellarein tetra-methylether		>100 μM	50% (IC50) [47]
Anti-angiogenic effect on zebrafish embryos	Sinensetin		30 μM	95% inhibited [47]
	Nobiletin		30 μM	63% inhibited [47]
	Hesperetin		30 μM	30% inhibited [47]

## Data Availability

Raw data supporting the conclusions of this article will be made available by the authors upon request due to the data are part of an ongoing study.

## Abbreviations

The following abbreviations are used in this manuscript:

4′-DN	4′-demethylnobiletin
4′-DT	4′-demethyltangeretin
AA	Arachidonic acid
AMPK	Adenosine monophosphate-activated protein kinase
AP-1	Activator protein-1
BACH1	BTB and CNC homology 1
BMD	Bone mineral density
BMM	Bone marrow macrophage
BMP	Bone morphogenetic protein
BV/TV	Bone volume/tissue volume
CCL	CC chemokine ligand
CIA	Collagen-induced arthritis
COX	Cyclooxygenase
CXCL	C-X-C motif chemokine ligand
DDS	Drug delivery system
DMM	Medial meniscus
EP	PGE_2_ receptor subtype
ERK	Extracellular signal-regulated kinase
FLS	Fibroblast-like synoviocyte
GPCR	G-protein coupled receptor
HMF	Heptamethoxyflavone
HO-1	Heme oxygenase-1
HUVEC	Human umbilical vein endothelial cell
IC50	50% inhibitory concentration
IKK	Inhibitor of NF-kappa B kinase
IL	Interleukin
iNOS	Inducible NO synthase
i.p.	Intraperitoneal
JNK	c-Jun N-terminal kinase
LPS	Lipopolysaccharide
MAPK	Mitogen-activated protein kinase
M-CSF	Macrophage colony-stimulating factor
MMP	Matrix metalloproteinase
mPGES-1	Membrane-type PGE synthase-1
mTOR	Mechanistic target of rapamycin
NFATc1	Nuclear factor of activated T cells 1
NF-κB	Nuclear factor-kappa B
NO	Nitric oxide
Nrf2	Nuclear factor erythroid 2-related factor 2
OA	Osteoarthritis
OPG	Osteoprotegerin
OVX	Ovariectomy
PG	Prostaglandin
PLA	Phospholipase A
PMF	Polymethoxyflavone
RA	Rheumatoid arthritis
RANKL	Receptor activator of NF-kappa B ligand
RASF	Rheumatoid synovial fibroblast
ROS	Reactive oxygen species
RUNX2	Runt-related transcription factor 2
SAR	Structure-biological activity relationship (structure-activity relationship)
SERPINA3	Serpin family A Member 3
TNF	Tumor necrosis factor

## References

[1] Okamoto K. (2024). Crosstalk between Bone and the Immune System. J. Bone Miner. Metab..

[2] Umur E., Bulut S.B., Yiğit P., Bayrak E., Arkan Y., Arslan F., Baysoy E., Kaleli-Can G., Ayan B. (2024). Exploring the Role of Hormones and Cytokines in Osteoporosis Development. Biomedicines.

[3] Takayanagi H. (2021). RANKL as the Master Regulator of Osteoclast Differentiation. J. Bone Miner. Metab..

[4] Huang Y.-S., Ho S.-C. (2010). Polymethoxy Flavones Are Responsible for the Anti-Inflammatory Activity of Citrus Fruit Peel. Food Chem..

[5] Zhang L., Zhang X., Zhang C., Bai X., Zhang J., Zhao X., Chen L., Wang L., Zhu C., Cui L. (2016). Nobiletin Promotes Antioxidant and Anti-Inflammatory Responses and Elicits Protection against Ischemic Stroke in Vivo. Brain Res..

[6] Jin Y.-J., Jang M.-G., Kim J.-W., Baek S., Ko H.-C., Hur S.-P., Kim S.-J. (2022). Anti-Obesity Effects of Polymethoxyflavone-Rich Fraction from Jinkyool (*Citrus sunki* Hort. Ex Tanaka) Leaf on Obese Mice Induced by High-Fat Diet. Nutrients.

[7] Harada S., Tominari T., Matsumoto C., Hirata M., Takita M., Inada M., Miyaura C. (2011). Nobiletin, a Polymethoxy Flavonoid, Suppresses Bone Resorption by Inhibiting NFκB-Dependent Prostaglandin E Synthesis in Osteoblasts and Prevents Bone Loss Due to Estrogen Deficiency. J. Pharmacol. Sci..

[8] Tominari T., Hirata M., Matsumoto C., Inada M., Miyaura C. (2012). Polymethoxy Flavonoids, Nobiletin and Tangeretin, Prevent Lipopolysaccharide-Induced Inflammatory Bone Loss in an Experimental Model for Periodontitis. J. Pharmacol. Sci..

[9] Matsumoto C., Inoue H., Tominari T., Watanabe K., Hirata M., Miyaura C., Inada M. (2014). Heptamethoxyflavone, a Citrus Flavonoid, Suppresses Inflammatory Osteoclastogenesis and Alveolar Bone Resorption. Biosci. Biotechnol. Biochem..

[10] Hirata M., Tominari T., Ichimaru R., Takiguchi N., Tanaka Y., Takatoya M., Arai D., Yoshinouchi S., Miyaura C., Matsumoto C. (2023). Effects of 4′-Demethylnobiletin and 4′-Demethyltangeretin on Osteoclast Differentiation In Vitro and in a Mouse Model of Estrogen-Deficient Bone Resorption. Nutrients.

[11] Wang B., Wu L., Chen J., Dong L., Chen C., Wen Z., Hu J., Fleming I., Wang D.W. (2021). Metabolism Pathways of Arachidonic Acids: Mechanisms and Potential Therapeutic Targets. Signal Transduct. Target. Ther..

[12] Ono T., Hayashi M., Sasaki F., Nakashima T. (2020). RANKL Biology: Bone Metabolism, the Immune System, and Beyond. Inflamm. Regen..

[13] Liu K., Wang Z., Liu J., Zhao W., Qiao F., He Q., Shi J., Meng Q., Wei J., Cheng L. (2023). Atsttrin Regulates Osteoblastogenesis and Osteoclastogenesis through the TNFR Pathway. Commun. Biol..

[14] Tominari T., Sanada A., Ichimaru R., Matsumoto C., Hirata M., Itoh Y., Numabe Y., Miyaura C., Inada M. (2021). Gram-Positive Bacteria Cell Wall-Derived Lipoteichoic Acid Induces Inflammatory Alveolar Bone Loss through Prostaglandin E Production in Osteoblasts. Sci. Rep..

[15] Tominari T., Akita M., Matsumoto C., Hirata M., Yoshinouchi S., Tanaka Y., Karouji K., Itoh Y., Maruyama T., Miyaura C. (2022). Endosomal TLR3 Signaling in Stromal Osteoblasts Induces Prostaglandin E2–Mediated Inflammatory Periodontal Bone Resorption. J. Biol. Chem..

[16] Suzawa T., Miyaura C., Inada M., Maruyama T., Sugimoto Y., Ushikubi F., Ichikawa A., Narumiya S., Suda T. (2000). The Role of Prostaglandin E Receptor Subtypes (EP1, EP2, EP3, and EP4) in Bone Resorption: An Analysis Using Specific Agonists for the Respective EPs. Endocrinology.

[17] Inada M., Matsumoto C., Uematsu S., Akira S., Miyaura C. (2006). Membrane-Bound Prostaglandin E Synthase-1-Mediated Prostaglandin E2 Production by Osteoblast Plays a Critical Role in Lipopolysaccharide-Induced Bone Loss Associated with Inflammation. J. Immunol..

[18] Attur M., Al-Mussawir H.E., Patel J., Kitay A., Dave M., Palmer G., Pillinger M.H., Abramson S.B. (2008). Prostaglandin E2 Exerts Catabolic Effects in Osteoarthritis Cartilage: Evidence for Signaling via the EP4 Receptor. J. Immunol..

[19] Sun Q., Zhang Y., Ding Y., Xie W., Li H., Li S., Li Y., Cai M. (2022). Inhibition of PGE2 in Subchondral Bone Attenuates Osteoarthritis. Cells.

[20] Markovič T., Jakopin Ž., Dolenc M.S., Mlinarič-Raščan I. (2017). Structural Features of Subtype-Selective EP Receptor Modulators. Drug Discov. Today.

[21] Miyaura C., Inada M., Suzawa T., Sugimoto Y., Ushikubi F., Ichikawa A., Narumiya S., Suda T. (2000). Impaired Bone Resorption to Prostaglandin E2 in Prostaglandin E Receptor EP4-Knockout Mice. J. Biol. Chem..

[22] Murakami A., Song M., Katsumata S., Uehara M., Suzuki K., Ohigashi H. (2007). Citrus Nobiletin Suppresses Bone Loss in Ovariectomized DdY Mice and Collagen-induced Arthritis in DBA/1J Mice: Possible Involvement of Receptor Activator of NF-kappaB Ligand (RANKL)-induced Osteoclastogenesis Regulation. BioFactors.

[23] Pang Y., Liu L., Mu H., Veeraraghavan V.P. (2021). Nobiletin Promotes Osteogenic Differentiation of Human Osteoblastic Cell Line (MG-63) through Activating the BMP-2/RUNX-2 Signaling Pathway. Saudi J. Biol. Sci..

[24] Rojasawasthien T., Usui M., Addison W.N., Matsubara T., Shirakawa T., Tsujisawa T., Nakashima K., Kokabu S. (2023). Nobiletin, a NF-κB Signaling Antagonist, Promotes BMP-induced Bone Formation. FASEB BioAdv..

[25] Lee Y.-S., Asai M., Choi S.-S., Yonezawa T., Teruya T., Nagai K., Woo J.-T., Cha B.-Y. (2014). Nobiletin Prevents Body Weight Gain and Bone Loss in Ovariectomized C57BL/6J Mice. Pharmacol. Pharm..

[26] Wang Y., Xie J., Ai Z., Su J. (2019). Nobiletin-Loaded Micelles Reduce Ovariectomy-Induced Bone Loss by Suppressing Osteoclastogenesis. Int. J. Nanomed..

[27] Hosokawa Y., Hosokawa I., Ozaki K. (2021). Nobiletin Decreases Inflammatory Mediator Expression in Tumor Necrosis Factor-Stimulated Human Periodontal Ligament Cells. Mediat. Inflamm..

[28] Hosokawa Y., Hosokawa I., Ozaki K., Matsuo T. (2021). Nobiletin Inhibits Inflammatory Reaction in Interleukin-1β-Stimulated Human Periodontal Ligament Cells. Pharmaceutics.

[29] Imada K., Lin N., Liu C., Lu A., Chen W., Yano M., Sato T., Ito A. (2008). Nobiletin, a Citrus Polymethoxy Flavonoid, Suppresses Gene Expression and Production of Aggrecanases-1 and -2 in Collagen-Induced Arthritic Mice. Biochem. Biophys. Res. Commun..

[30] Liu Z., Guo S., Dong Q. (2020). Nobiletin Suppresses IL-21/IL-21 Receptor-Mediated Inflammatory Response in MH7A Fibroblast-like Synoviocytes (FLS): An Implication in Rheumatoid Arthritis. Eur. J. Pharmacol..

[31] Xie L., Xie H., Chen C., Tao Z., Zhang C., Cai L. (2019). Inhibiting the PI3K/AKT/NF-ΚB Signal Pathway with Nobiletin for Attenuating the Development of Osteoarthritis: In Vitro and in Vivo Studies. Food Funct..

[32] Lin Z., Wu D., Huang L., Jiang C., Pan T., Kang X., Pan J. (2019). Nobiletin Inhibits IL-1β-Induced Inflammation in Chondrocytes via Suppression of NF-ΚB Signaling and Attenuates Osteoarthritis in Mice. Front. Pharmacol..

[33] Li Y.-J., Zhang T., Tu J.-X., Li G., Zhou Y. (2015). Tangeretin Inhibits IL-1? Induced Proliferation of Rheumatoid Synovial Fibroblasts and the Production of COX-2, PGE2 and MMPs via Modulation of P38 MAPK/ERK/JNK Pathways. Bangladesh J. Pharmacol..

[34] Li X., Xie P., Hou Y., Chen S., He P., Xiao Z., Zhan J., Luo D., Gu M., Lin D. (2019). Tangeretin Inhibits Oxidative Stress and Inflammation via Upregulating Nrf-2 Signaling Pathway in Collagen-Induced Arthritic Rats. Pharmacology.

[35] Yuan Z., Li J., Xiao F., Wu Y., Zhang Z., Shi J., Qian J., Wu X., Yan F. (2024). Sinensetin Protects against Periodontitis through Binding to Bach1 Enhancing Its Ubiquitination Degradation and Improving Oxidative Stress. Int. J. Oral Sci..

[36] Liu Z., Liu R., Wang R., Dai J., Chen H., Wang J., Li X. (2022). Sinensetin Attenuates IL-1β-Induced Cartilage Damage and Ameliorates Osteoarthritis by Regulating SERPINA3. Food Funct..

[37] Zhou W., Shi Y., Wang H., Yu C., Zhu H., Wu A. (2021). Sinensetin Reduces Osteoarthritis Pathology in the Tert-Butyl Hydroperoxide-Treated Chondrocytes and the Destabilization of the Medial Meniscus Model Mice via the AMPK/MTOR Signaling Pathway. Front. Pharmacol..

[38] Manthey J.A., Bendele P. (2008). Anti-Inflammatory Activity of an Orange Peel Polymethoxylated Flavone, 3′,4′,3,5,6,7,8-Heptamethoxyflavone, in the Rat Carrageenan/Paw Edema and Mouse Lipopolysaccharide-Challenge Assays. J. Agric. Food Chem..

[39] Okuyama S., Miyoshi K., Tsumura Y., Amakura Y., Yoshimura M., Yoshida T., Nakajima M., Furukawa Y. (2015). 3,5,6,7,8,3′,4′-Heptamethoxyflavone, a Citrus Polymethoxylated Flavone, Attenuates Inflammation in the Mouse Hippocampus. Brain Sci..

[40] Matsumoto S., Tominari T., Matsumoto C., Yoshinouchi S., Ichimaru R., Watanabe K., Hirata M., Grundler F.M.W., Miyaura C., Inada M. (2018). Effects of Polymethoxyflavonoids on Bone Loss Induced by Estrogen Deficiency and by LPS-Dependent Inflammation in Mice. Pharmaceuticals.

[41] Ohyama Y., Ito J., Kitano V.J., Shimada J., Hakeda Y. (2018). The Polymethoxy Flavonoid Sudachitin Suppresses Inflammatory Bone Destruction by Directly Inhibiting Osteoclastogenesis Due to Reduced ROS Production and MAPK Activation in Osteoclast Precursors. PLoS ONE.

[42] Hosokawa Y., Hosokawa I., Ozaki K., Matsuo T. (2019). Sudachitin Inhibits Matrix Metalloproteinase-1 and -3 Production in Tumor Necrosis Factor-α-Stimulated Human Periodontal Ligament Cells. Inflammation.

[43] Tsai Y.-M., Chong I.-W., Hung J.-Y., Chang W.-A., Kuo P.-L., Tsai M.-J., Hsu Y.-L. (2015). Syringetin Suppresses Osteoclastogenesis Mediated by Osteoblasts in Human Lung Adenocarcinoma. Oncol. Rep..

[44] Li S., Sang S., Pan M.-H., Lai C.-S., Lo C.-Y., Yang C.S., Ho C.-T. (2007). Anti-Inflammatory Property of the Urinary Metabolites of Nobiletin in Mouse. Bioorg. Med. Chem. Lett..

[45] Wang M., Meng D., Zhang P., Wang X., Du G., Brennan C., Li S., Ho C.-T., Zhao H. (2018). Antioxidant Protection of Nobiletin, 5-Demethylnobiletin, Tangeretin, and 5-Demethyltangeretin from Citrus Peel in Saccharomyces Cerevisiae. J. Agric. Food Chem..

[46] Kawaii S., Ikuina T., Hikima T., Tokiwano T., Yoshizawa Y. (2012). Relationship between Structure and Antiproliferative Activity of Polymethoxyflavones towards HL60 Cells. Anticancer. Res..

[47] Lam I.K., Alex D., Wang Y., Liu P., Liu A., Du G., Lee S.M.Y. (2012). In Vitro and in Vivo Structure and Activity Relationship Analysis of Polymethoxylated Flavonoids: Identifying Sinensetin as a Novel Antiangiogenesis Agent. Mol. Nutr. Food Res..

[48] Yoshino S., Tagawa T., Awa R., Ogasawara J., Kuwahara H., Fukuhara I. (2021). Polymethoxyflavone Purified from *Kaempferia parviflora* Reduces Visceral Fat in Japanese Overweight Individuals: A Randomised, Double-Blind, Placebo-Controlled Study. Food Funct..

[49] Yamada S., Shirai M., Ono K., Teruya T., Yamano A., Woo J.T. (2021). Beneficial Effects of a Nobiletin-rich Formulated Supplement of Sikwasa (*C. depressa*) Peel on Cognitive Function in Elderly Japanese Subjects; A Multicenter, Randomized, Double-blind, Placebo-controlled Study. Food Sci. Nutr..

[50] Ilieș M., Uifălean A., Pașca S., Dhople V.M., Lalk M., Iuga C.A., Hammer E. (2020). From Proteomics to Personalized Medicine: The Importance of Isoflavone Dose and Estrogen Receptor Status in Breast Cancer Cells. J. Pers. Med..

[51] Chen J., Chen A.Y., Huang H., Ye X., Rollyson W.D., Perry H.E., Brown K.C., Rojanasakul Y., Rankin G.O., Dasgupta P. (2015). The Flavonoid Nobiletin Inhibits Tumor Growth and Angiogenesis of Ovarian Cancers via the Akt Pathway. Int. J. Oncol..

[52] Sp N., Kang D.Y., Joung Y.H., Park J.H., Kim W.S., Lee H.K., Song K.-D., Park Y.-M., Yang Y.M. (2017). Nobiletin Inhibits Angiogenesis by Regulating Src/FAK/STAT3-Mediated Signaling through PXN in ER^+^ Breast Cancer Cells. Int. J. Mol. Sci..

[53] Wei D., Zhang G., Zhu Z., Zheng Y., Yan F., Pan C., Wang Z., Li X., Wang F., Meng P. (2019). Nobiletin Inhibits Cell Viability via the SRC/AKT/STAT3/YY1AP1 Pathway in Human Renal Carcinoma Cells. Front. Pharmacol..

[54] Xiao B., Liu Y., Chandrasiri I., Adjei-Sowah E., Mereness J., Yan M., Benoit D.S.W. (2024). Bone-Targeted Nanoparticle Drug Delivery System-Mediated Macrophage Modulation for Enhanced Fracture Healing. Small.

[55] Zhao R., Chen S., Yuan B., Chen X., Yang X., Song Y., Tang H., Yang X., Zhu X., Zhang X. (2019). Healing of Osteoporotic Bone Defects by Micro-/Nano-Structured Calcium Phosphate Bioceramics. Nanoscale.

[56] Farrell K.B., Karpeisky A., Thamm D.H., Zinnen S. (2018). Bisphosphonate Conjugation for Bone Specific Drug Targeting. Bone Rep..

